# Neural regulation of mesenchymal stem cells in craniofacial bone: development, homeostasis and repair

**DOI:** 10.3389/fphys.2024.1423539

**Published:** 2024-07-29

**Authors:** Huai-Jin Pi, Bo Huang, Quan Yuan, Jun-Jun Jing

**Affiliations:** State Key Laboratory of Oral Diseases, National Center for Stomatology, National Clinical Research Center for Oral Diseases, West China Hospital of Stomatology, Sichuan University, Chengdu, China

**Keywords:** craniofacial bone, mesenchymal stem cell, nerve, development, homeostasis, repair

## Abstract

Mesenchymal stem cells endow various functions, including proliferation, multipotency, migration, etc. Craniofacial bones originate from the cranial neural crest and are developed mainly through intramembranous ossification, which are different from long bones. There are varied mesenchymal stem cells existing in the craniofacial bone, including Gli1 + cells, Axin2 + cells, Prx1 + cells, etc. Nerves distributed in craniofacial area are also derived from the neural crest, and the trigeminal nerve is the major sensory nerve in craniofacial area. The nerves and the skeleton are tightly linked spatially, and the skeleton is broadly innervated by sensory and sympathetic nerves, which also participate in bone development, homeostasis and healing process. In this review, we summarize mesenchymal stem cells located in craniofacial bone or, to be more specific, in jaws, temporomandibular joint and cranial sutures. Then we discuss the research advance concerning neural regulation of mesenchymal stem cells in craniofacial bone, mainly focused on development, homeostasis and repair. Discovery of neural regulation of mesenchymal stem cells may assist in treatment in the craniofacial bone diseases or injuries.

## 1 Introduction

The concept of mesenchymal stem cell (MSC) suggests the existence of stem and/or progenitor cell populations in adult and neonatal tissues, which possess the capacity of providing de novo-specialized cells of mesodermal lineage (Naji et al., 2019). MSCs are able to differentiate into adipocytes, chondroblasts and osteoblasts under a definite stimulation *in vitro* (Naji et al., 2019). Typically, MSCs are derived from adult bone marrow and adipose tissue, and neonatal tissue such as umbilical cord is also utilized to obtain MSCs (Hass et al., 2011). Among the different sources of MSCs, bone marrow remains the most valued since it is better documented and widely used in preclinical and clinical studies (Yin et al., 2019). The function of MSCs include proliferation, multipotency, trophic ability, homing/migration and immunosuppression in fundamental aspects and their clinical implications (Naji et al., 2019).

The skeleton, comprised of specialized connective tissues, includes ossified/non-ossified elements, bone marrow stroma, and supportive tissues (Karsenty, 2003). These tissues are generated by diverse cell types, including osteocytes, chondrocytes, hematologic and stromal cells (Chan et al., 2015; Chan et al., 2018). Craniofacial bones are different from long bones concerning their developmental origins and osteogenic programs and structures (Chai et al., 2000). Long bones originate from trunk mesoderm, whereas most craniofacial bones are derived from the cranial neural crest (Chai et al., 2000). Craniofacial bones are flat and they are developed mainly through intramembranous ossification rather than endochondral ossification (Chai et al., 2000). In endochondral ossification, a cartilaginous template is formed, and then cartilage is gradually replaced with bone (Berendsen and Olsen, 2015). In intramembranous ossification, no cartilage is observed, and bony tissue is directly formed (Berendsen and Olsen, 2015). It should be noted that temporomandibular joint (TMJ) condyle undergoes endochondral ossification in the developmental stage (Stocum and Roberts, 2018).

Nerves distributed in craniofacial area are also derived from neural crest cells. During the migration, neural crest cells experience an epithelial-mesenchymal transition and differentiate into a wide range of derivatives, including neurons of the dorsal root ganglia of spinal nerves, some neurons of cranial ganglia, the autonomic nervous system (both sympathetic and parasympathetic ganglia) and Schwann cells which are responsible for the myelination of peripheral nerves (La Noce et al., 2014; Soldatov et al., 2019). The trigeminal nerve is the major sensory nerve in craniofacial area. The trigeminal ganglion (sensory branch) divides into three subdivisions, including ophthalmic (V1), maxillary (V2), and mandibular (V3) nerves (Freddi et al., 2022). Inferior alveolar nerve (IAN) is one of the branches of V3, which enters the mandibular foramen at the lingual surface of the mandibular ramus, travels within the inferior alveolar canal and exits through the mental foramen giving off the mental nerve (Freddi et al., 2022).

The nerves and the skeleton are tightly linked spatially, and the skeleton is broadly innervated by sensory and sympathetic nerves (Xu et al., 2022b). The nervous system’s role in regulating bone development, metabolism, homeostasis and remodeling has been confirmed (Assefa, 2023). However, our knowledge of the interaction between nervous system and skeletal system is still inadequate (Xu et al., 2022b). How the nerves regulate bone homeostasis suggests a brand-new avenue of research in bone biology (Wang et al., 2021). To date, researchers have been exploring neural regulation of stem cells in the skeletal microenvironment, gradually uncovering the roles of nerves in bone development, homeostasis and remodeling (Grässel, 2014; Chen et al., 2019; Wang et al., 2020a; Tomlinson et al., 2020). Nerve fibers are discovered to be involved in the formation of the bone marrow stem cell niche (Méndez-Ferrer et al., 2010).

In this review article, we list and delineate MSCs existing in jaws, TMJ and cranial sutures. Then we discuss the role of nervous system in craniofacial bone via the regulation of MSCs, mainly focused on development, homeostasis and repair, and hopefully shedding new light on modulation and treatment in the bone diseases and injuries.

## 2 MSCs in craniofacial bone

**FIGURE 1:**
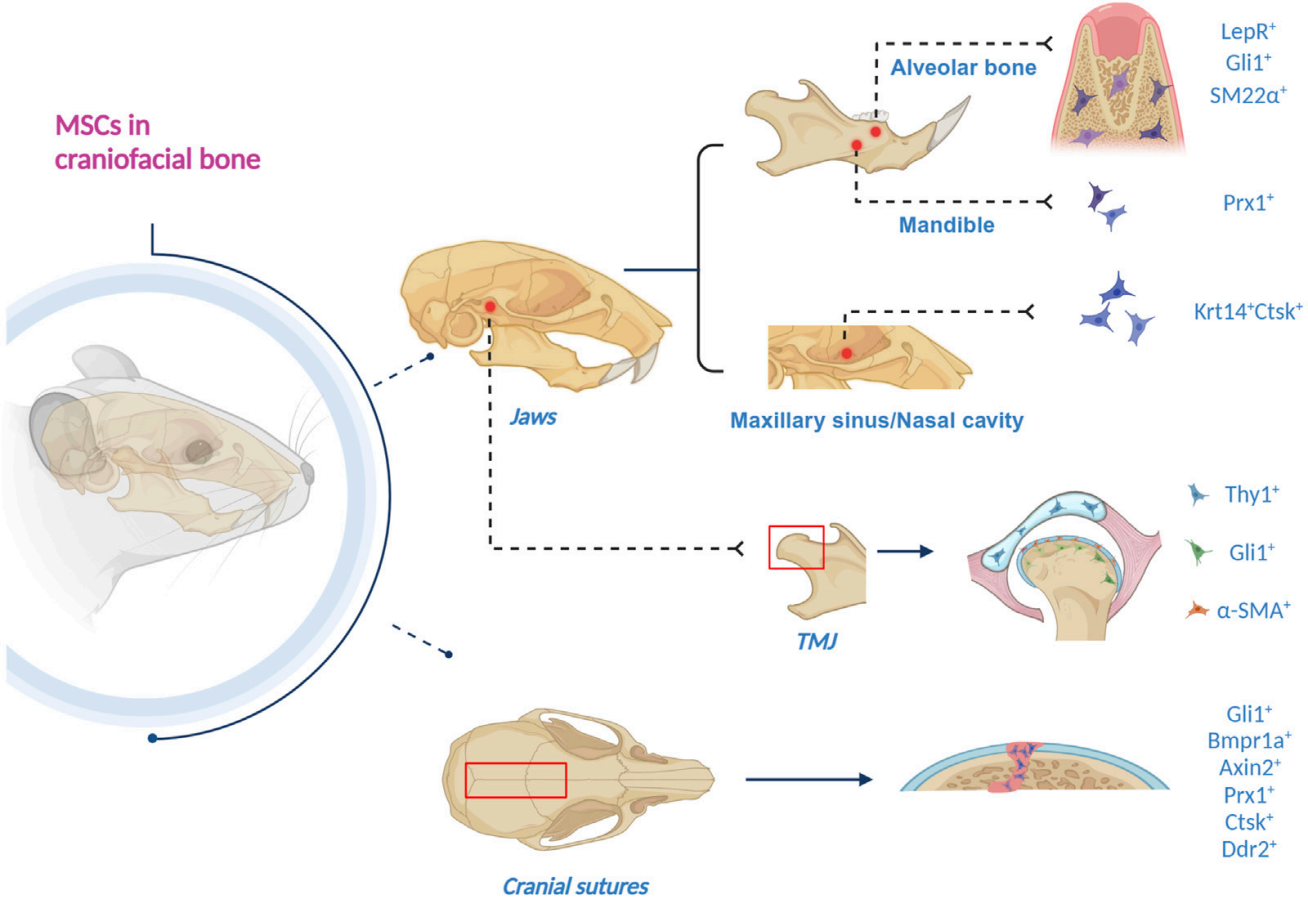
Schematics of craniofacial MSCs, residing in jaws, TMJ and cranial sutures. 1) In the alveolar bone, LepR+, Gli1+, and SM22α + MSCs have been discovered. 2) Gli1 + MSCs also reside in apical mesenchyme. 3) Prx1 + MSCs exist in the mandible. 4) Krt14 + Ctsk + MSCs are in the maxillary sinus and nasal cavity. 5) Gli1 + MSCs are in the TMJ subchondral bone, the periosteum and the perichondrium of TMJ condyle. 6) α-SMA + MSCs reside in the fibrous superficial zone tissue in the TMJ condyle. 7) Thy1 + MSCs are in the TMJ disc. 8) Gli1+, Bmpr1a+, Axin2+, Prx1+, Ctsk + and Ddr2 + MSCs have been found in the cranial sutures (Created with BioRender.com).

MSCs in craniofacial bones are diversified and display a series of markers. They primarily reside in jaws, TMJ and cranial sutures (Figure 1).

### 2.1 MSCs in jaws

Unlike long bones, our understanding of stem cells in jaws is insufficient (Li et al., 2022a). To date, seven markers labeling MSC residing in the jaws have been discovered, i.e., LepR, Gli1, SM22α, Fat4, Prx1, and Krt14/Ctsk.

Research on long bones has revealed that leptin receptor (LepR)-expressing bone marrow stromal cells are the major source of osteoprogenitor cells, which arose postnatally and differentiated into most bone and adipocytes (Zhou et al., 2014). Zhang et al. (2020a) identified tissue-resident LepR + MSCs in alveolar bone of the adult mice, which maintained in a quiescent state but rapidly got activated upon injury and were differentiated into osteoblasts. Further studies indicated that LepR + MSCs are indispensable for a normal jaw bone-healing process, in which PTH/PTH1R signal axis plays a critical role (Zhang et al., 2020a).

Gli1 + cells have been identified as stem cells in many tissues (Zhao and Chai, 2015; Schneider et al., 2017; Sena et al., 2017; Shi et al., 2017). Gli1 is a direct target gene of hedgehog signaling, and Indian hedgehog and Gli1 play a pivotal role in the regulation of craniofacial development (Sun et al., 2024). It has been discovered that Gli1 + cells surrounding the neurovascular bundle are MSCs that support the homeostasis and repair of incisor mesenchyme (Zhao et al., 2014). In addition, Gli1 + cells were identified as MSCs in the developing molar apical mesenchyme in a mouse model (Feng et al., 2017). Yi et al. (2022) found Gli1 + cells residing in alveolar bone marrow (ABM), and with the assistance of lineage tracing, they discovered that Gli1 + cells in ABM are activated and proliferate along blood vessels following tooth extraction, contributing to socket healing and implant osseointegration.

SM22α is primarily expressed in mural cells, visceral smooth muscles and myofibroblasts, and the roles of SM22α + cells in niches of tissue development and tumor malignancy have been clarified (Zhang et al., 2020b; Muhl et al., 2020; Li et al., 2021). In alveolar bone, SM22α-lineage niche cells are activated upon injury (Zhou et al., 2022). Interestingly, SM22α-lineage niche cells did not exhibit MSC-like expression pattern, and instead regulated various processes of alveolar bone regeneration via PDGFRβ-driven H₂S generation (Zhou et al., 2022).

Utilizing single-cell RNA sequencing (scRNA-seq), Jin et al. (2023) uncovered a previously unidentified cell population exhibiting a high expression of protocadherin Fat4, which is specifically enriched around ABM cavities. It was demonstrated that Fat4 + cells possess colony-forming, osteogenic, and adipogenic capabilities, with the core transcriptional features regulating osteogenic differentiation, verifying that these cells belong to MSCs residing in the alveolar bone (Jin et al., 2023).

Prx1 is a homeobox gene expressed in the skeleton system, and its mutation has been discovered to be related to underdeveloped mandible (Esposito et al., 2020). A recent study has elucidated the crucial role of Prx1-expressing cells as stem cells for bone, which is mainly reflected in skeletal development, growth, homeostasis and aging (Liu et al., 2022). Jiang et al. established canine distraction osteogenesis (DO) models of the mandible, revealing the reversion of Prx1+MSCs to primitive neural-crest-cell-like state (Jiang et al., 2023). In addition, this study provided evidence demonstrating that Prx1 deficiency leads to impaired osteogenic ability in DO (Jiang et al., 2023).

A subset of cells co-expressing epithelial (Krt14) and mesenchymal (Ctsk) markers in maxillary sinus and the nasal cavity have been identified as osteoprogenitors, contributing to homeostasis and injury-induced osteogenesis of the maxillary sinus floor (Weng et al., 2022). Krt14 + Ctsk + cells exhibit both epithelial and mesenchymal properties, and specifically play a role in bone regeneration after maxillary sinus floor lifting (Weng et al., 2022). Lineage tracing with dual recombinases showed that descendants of Krt14 + Ctsk + progenitors, which are Krt14-Ctsk+, underwent robust osteogenesis, revealing Krt14 + Ctsk + cells serve as a novel lineage of osteoprogenitor cells in craniofacial bone regeneration (Weng et al., 2022).

### 2.2 MSCs in TMJ

The temporomandibular joint (TMJ) is a unique articulation of the craniofacial bone between the mandible and the temporal bone. The main components of TMJ are articular condyle, articular disc, articular fossa, fibrous capsule, and synovial membrane (Palla, 2016). TMJ develops from three separate mesenchymal condensations representing the glenoid fossa of the temporal bone, the condyle of the mandibular ramus, and the articular disc (Stocum and Roberts, 2018). The active growth element of the condyle is a secondary cartilage derived from the medial periosteal cells expressing tenascin-C of the mandibular ramus, which will undergo endochondral ossification. So far, three markers have been discovered that label the stem cells in the TMJ region, i.e., Gli1, α-SMA and Thy1.

Utilizing lineage tracing analysis, Gli1 + cells were found residing in the subchondral bone, serving as osteogenic progenitors for postnatal osteogenesis and homeostasis in the mouse mandibular condyle (Lei et al., 2022). Activation of Gli1 + cells contributes to increased and uneven distribution of osteoblastic lineage differentiation, interfering the homeostasis of subchondral bone region and accelerating pathological changes during the development of TMJ osteoarthritis (Lei et al., 2022). In another study, it was revealed that Gli1 + cells reside within both the periosteum and the perichondrium of condyle (Zhang et al., 2023a). Further, the condylar Gli1 + cells possess both osteogenic and chondrogenic potential while the periosteal-associated cells only have osteogenic potential (Zhang et al., 2023a).

In these years, a few studies have indicated that MSC population resides in the articular cartilage, playing a critical role in cell renewal and differentiation during cartilage modeling and repair (Tong et al., 2015; Levato et al., 2017; Wang et al., 2020b). Embree et al. discovered that the fibrous superficial zone tissue in the TMJ condyle is a niche harboring fibrocartilage stem cells (FCSCs), which were first isolated from the rat TMJ (Embree et al., 2016). Utilizing lineage tracing analysis, mouse FCSCs in the TMJ condyle were identified with the stem cell label α-SMA (Embree et al., 2016). FCSCs were found to possess the capacity of cartilage regeneration and repair upon injury (Embree et al., 2016).

It should be noted that Bi et al. (2020) verified a stem cell population residing in the superficial layer of human TMJ condylar cartilage, named human fibrocartilage stem cells (hFCSCs), which exhibit distinct stemness. In addition, research results from Yin et al. (2023) highlight that hFCSCs from condyle osteochondroma displayed a specific chondrogenic differentiation capacity and were liable to forming more mature and hypertrophic cartilage during the chondrogenic inducing process. Besides, abnormally activated PI3K/AKT signaling in condyle osteochondroma has the potential for leading to functional changes of hFCSCs by regulating cell apoptosis, bringing about TMJ cartilage lesion in condyle osteochondroma patients or animals (Yin et al., 2023). Nevertheless, no specific marker has been identified so far to label hFCSCs.

In order to deepen the understanding of cellular composition of TMJ discs, a comprehensive scRNA-seq analysis of mouse TMJ disc tissues was performed and four principal cell types were identified, which exhibited various transcript expression characteristics and cellular functions, including fibroblast, macrophage, endothelial cell and mural cell (Bi et al., 2023). It was confirmed that Thy1 + mural cells in TMJ articular discs manifest significantly stronger stem cell characteristics than other Thy1-disc cells *in vitro* and *ex vivo*, such as higher colony formation potential as well as proliferative capacities (Bi et al., 2023). Moreover, in virtue of tri-lineage transduced differentiation *in vitro*, Thy1 + cells were also found to possess markedly stronger potential for osteogenesis, adipogenesis and chondrogenesis (Bi et al., 2023). Mural cells, including Thy1 + cells, were demonstrated to transform toward non-chondrogenic fibroblasts in TMJ disc injury (Bi et al., 2023).

### 2.3 MSCs in cranial sutures

Cranial sutures have been identified as the major location of MSCs, revealing that the regeneration capacity of the craniofacial bones should reside within the sutures (Park et al., 2016). It has been found that sutures possess much stronger regeneration capacity than other regions of the calvaria (Park et al., 2016). So far, there are six markers verified to be labelled suture mesenchymal stem cells (SuSCs), including Gli1, Axin2, Bmpr1a, Prx1, Ctsk and Ddr2.

Gli1+ cells within the suture mesenchyme were first verified as the main SuSCs for adult craniofacial bones by Zhao et al. (2015). Assisted by lineage tracing analysis, this team delineated that Gli1 + SuSCs in the suture give rise to osteogenic fronts, periosteum and dura (Zhao et al., 2015). Subsequently, Park et al. (2016) further confirmed that Gli1 + SuSCs within the suture mesenchyme are the cellular source for injury repair and bone regeneration, emphasizing that bone healing capacity of calvaria is primarily restricted in sutures rather than evenly distributed on the calvarial surface. Another research established rapid maxillary expansion mouse models and mechanical stretch loading cell models, and it was uncovered that Gli1 + cells in maxillofacial sutures participate in mechanical force-induced bone formation (Huang et al., 2021). Additionally, Jing et al. (2022) utilized a standardized model for mouse suture expansion and first demonstrated that Gli1 + SuSCs are the stem cell source to facilitate craniofacial DO, in which canonical Wnt signaling expressed in Gli1 + SuSCs is essential.

Maruyama et al. (2016) first identified Axin2 + cells as SuSCs, providing solid evidence to prove their stemness, including longterm self-renewing, clonal expanding and differentiating abilities, and demonstrated their injury repair and regenerative capacity. It is important to note that unlike Gli1 + SuSCs, Axin2 + SuSCs are mainly located and concentrated in the midline of the suture mesenchyme at early stages after birth (Maruyama et al., 2016). Lineage tracing analysis suggested that Axin2 + SuSCs and their derivatives are widely distributed in all patent sutures and calvarial bones over a long tracing period (Maruyama et al., 2016). Gene-profiling analysis illustrated high levels of Gli1 expression in the Axin2-expressing cells, which implied an overlap of these two cell populations (Maruyama et al., 2016). It can be assumed that Axin2 + SuSCs likewise possess the potential for bone regeneration and injury repair.

Maruyama et al. (2021) conducted further research and discovered BMP ligands signal via the type Ⅰ receptor Bmpr1a to activate the pathway in Axin2 + SuSCs. They demonstrated that Bmpr1a plays a crucial role in the maintenance of SuSC stemness, and regulates not only SuSC self-renewal but also SuSC-mediated osteogenesis (Maruyama et al., 2021). It should be noted that in both mice and humans, Bmpr1a/BMPR1A functions as an SuSC marker, and Bmpr1a regulates stem cell stemness, which is critical for suture patency and craniosynostosis (Maruyama et al., 2021).

Postnatal cells expressing Prx1 of the calvarial sutures have been verified exhibiting qualities of MSCs, and calvarial sutures proved to be the exclusive niche of Prx1 + cells (Wilk et al., 2017). Prx1 + SuSCs proved residing exclusively in the posterior frontal, coronal, sagittal, and lambdoid sutures, whereas they were not detectable in other craniofacial sutures, the calvarial periosteum or the dura mater (Wilk et al., 2017). This team confirmed that Prx1 + SuSCs and their progeny contribute to the development of calvarial sutures and calvarial bones, and play a role in calvarial bone defect regeneration (Wilk et al., 2017). Interestingly, this team presumed that Prx1 + SuSCs are a subpopulation of Axin2 + SuSCs (Wilk et al., 2017).

Debnath et al. (2018) identified a periosteal stem cell expressing Ctsk located in the long bones and calvaria of mice, and provided evidence of its clonal multipotency and self-renewal. Contrary to other MSCs which mediate endochondral ossification, Ctsk + stem cells choose intramembranous pathway for bone formation (Debnath et al., 2018). Ctsk + cells were discovered to acquire endochondral bone formation capacity in response to injury (Debnath et al., 2018). Subsequently, this team focused on craniosynostosis and calvarial mineralization, and they demonstrated the interaction of two separate stem cell lineages in the sutures: Ctsk + SuSCs and Ddr2 + SuSCs, which were first verified (Bok et al., 2023). Research data indicated that Ddr2 + SuSCs mediate a third fundamental form of bone formation, endochondral ossification with no haematopoiesis, alongside traditional intramembranous and endochondral ossification (Bok et al., 2023). It was revealed that interactions between these two cell types are essential for the pathogenesis of craniosynostosis and suture patency (Bok et al., 2023).

MSCs in craniofacial bone, despite different positions and diverse markers listed above, exhibit not only the capacity for long-term self-renewal, clonal expansion, differentiation and multipotency, but also the ability to respond to injuries and facilitate tissue regeneration.

## 3 Neural regulation of MSCs in craniofacial bone

The skeleton is mainly innervated by sensory and sympathetic nerves, and long bones and craniofacial bone are innervated by nerves originating at different locations (Xu et al., 2022b). Nerves play essential roles during the development of craniofacial tissues such as the salivary glands, teeth, and calvarial bones (Adameyko and Fried, 2016). The role of nervous system in bone homeostasis has been gradually realized in recent years (Chen et al., 2019; Meyers et al., 2020). Since nerves turned out to be involved in the stem cell microenvironment, the regulation of MSCs by the nervous system has become an increasing focus of research (Méndez-Ferrer et al., 2010; Zhao et al., 2014; Park et al., 2015; Maryanovich et al., 2018). Sensory and sympathetic nerves induce bone marrow MSCs (BMMSCs) migration to the osteogenesis front line through various neurotransmitters or neuropeptides, thus sustaining an active bone environment in osteogenesis (Wang et al., 2020a). In this section, we will discuss the role of neural regulation of MSCs in craniofacial bone development, homeostasis and repair.

### 3.1 Neural regulation of MSCs in craniofacial bone development

Research concerning neural regulation of MSCs in craniofacial bone development is still in its infancy, yet this topic is attracting increasing attention. Various cells, molecules or signaling pathways participate in this process, including Schwann cells, tropomyosin receptor kinase A (TrkA), FGF-SHH signaling, etc. Cranial bones, suture and teeth are the craniofacial elements demonstrated to be involved in the neural regulation.

Schwann cells and Schwann cell precursors (SCPs) were proved to be inactive neural-crest-like cells, which can be recruited from nerves, giving rise to dental MSCs and generating pulp cells and odontoblasts in adult teeth (Kaukua et al., 2014). It was further revealed that some SCPs detach from nerve fibers to become MSCs, contributing to chondrogenesis and osteogenesis (Xie et al., 2019). This process was demonstrated to appear exclusively during murine embryonic development, resulting in the formation of craniofacial elements without contribution to the development of the appendicular skeleton (Xie et al., 2019).

Gadomski et al. (2022) conducted research on mouse cranial and femoral bones and found that skeletal sympathetic cholinergic nerve fibers induced by interleukin-6 preserve osteocytes survival and function during postnatal development via a neurotrophic axis, which is mediated through GDNF (glial cell derived neurotrophic factor)-family receptor-α2 (GFRα2) and its ligand, neurturin (NRTN). *GFRa2* ^−/−^ mice showed reduced skeletal stem cells and bone formation, resulting in decreased bone mass and strength, enlarged cranial sutures and flatter skulls (Gadomski et al., 2022).

Tower et al. (2021) cast light on the role of TrkA + sensory nerves in suture patency of developing cranium. Research data clarified that TrkA signaling in peripheral afferent neurons is needed to sustain MSCs in sutures in a proliferative, undifferentiated state, and the loss of suture mesenchyme patency with a decline in cell proliferation contributes to the premature suture closure, leading to the failure of normal cranial vault expansion (Tower et al., 2021).

Fibroblast growth factor (FGF) plays a role in sensory nerves’ regulation of MSCs in craniofacial elements development, and it serves as a bridge connecting sensory nerves to Gli1 + MSCs. FGFs have been found to control a variety of physiological responses during embryonic development and in adult organisms, participating in patterning and morphogenesis via regulating cell survival, proliferation, migration and differentiation (Korsensky and Ron, 2016). Pei et al. (2024) discovered that FGF signaling modulates Gli1 + cells through its receptor FGFR2 during tooth root development in a mouse molar model, and FGF-SHH signaling axis participates in the regulation of tooth root morphogenesis. Loss of FGF signaling in Gli1 + cells results in shortened tooth roots with compromised root progenitor cell proliferation and differentiation (Pei et al., 2024).

The significance of neural regulation of MSCs in craniofacial bone development has been gradually uncovered. Nevertheless, except for the elements mentioned above, whether or how the development of other areas in the craniofacial bones is regulated by the nervous system requires further exploration.

### 3.2 Neural regulation of MSCs in craniofacial bone homeostasis and repair

#### 3.2.1 Neural regulation in jaws

Jaws are the research hotspot representing neural regulation of MSCs in craniofacial bone homeostasis and repair, and sensory nerves are the major participants. Inferior alveolar nerve (IAN) is one of the principal sensory nerves in the mandible, and numerous studies revealed that IAN denervation results in disorders of bone homeostasis and repair. Wu et al. (2016b) showed that IAN transection results in mandibular bone loss in an age-dependent way. Hayano et al. (2018) demonstrated that mesenchymal stem/progenitor cell is related to IAN innervation, and its denervation leads to the impairment of incisor growth and formation, or specifically, a reduction in incisor eruption and enamel defects. Jones et al. (2019) showed that after IAN denervation, mandibular bone repair is impaired due to the functional defects in MSCs, which can be partially rescued by Schwann cell transplantation and by Schwann-derived growth factors platelet-derived growth factor-AA, oncostatin M and parathyroid hormone. Tevlin et al. (2023) discovered that mandibular DO with IAN denervation results in impaired osteogenesis and reduced MSC amplification and osteogenic potential in mice, which are arguments for the connection between nerves and mandible repair. Additionally, a human patient specimen was analyzed in this research and indicated that the histological, radiological, and transcriptional changes seen in the mouse DO model may be consistent in the context of denervated human mandibular DO (Tevlin et al., 2023). These studies *in vivo* demonstrated the point that the sensory nerve denervation influences stem cells, disturbing craniofacial bone homeostasis and repair.

Several nerve-related molecules have been confirmed to participate in sensory nerves’ regulation of MSCs in jaws homeostasis and repair, such as Calcitonin gene-related peptide (CGRP), Substance P (SP) and Semaphorin 3A (Sema 3A). CGRP is a polypeptide with 37 amino acids (Russell et al., 2014), and it is mainly synthesized in sensory nerve fibers (Choi and Di Nardo, 2018), stored and released as vesicles (Arkless et al., 2019). CGRP is secreted from the trigeminal semilunar neuron and transported to the bone in the maxillofacial region, performing its biological effects via binding to the main receptor transient receptor potential vanilloid 1 (TRPV1) (Grässel, 2014). It was revealed that innervation improves the osteogenic capacity of BMMSCs via CGRP to activate p38 MAPK and Wnt6/β-catenin pathways, thereby maintaining bone homeostasis (Wu et al., 2023b). Two independent groups have demonstrated that inhibition of CGRP impairs long bone fracture healing and regeneration (Appelt et al., 2020; Wee et al., 2023). Except for long bones, CGRP also makes contribution to jaws homeostasis and repair. Pang et al. studied the expression patterns of CGRP and SHH signaling in the tooth socket and deduced that in the socket healing process following tooth extraction, CGRP released by sensory nerves in the periodontal tissue stimulates MSCs’ proliferation and osteogenic differentiation, enhances osteoblast activation and attenuates osteoclasts’ function, hence facilitating osteogenesis and restricting bone resorption, which were found to be mediated by SHH signaling (Pang et al., 2015). Liu et al. (2018b) showed that IAN neurectomy erodes osteogenesis surrounding titanium implants with less CGRP fluorescence in the immunofluorescence assay, yet the consequence can be partially balanced via the injection of CGRP around the implant *in vivo*, in a process possibly mediated by canonical Wnt signaling pathway. Additionally, a research group have conducted a series of studies on the role of biodegradable magnesium implant in bone tissue regeneration and its connection with CGRP (Zhang et al., 2016; Tian et al., 2018; Ye et al., 2021). Recently, the significance of CGRP in the function of periodontal ligament cells has been firstly reported. It was indicated that CGRP stimulation induces the differentiation of a mouse periodontal ligament cell line (MPDL22 cells) into hard tissue-forming cells, underscoring the critical role of CGRP signaling in alveolar bone repair (Miki et al., 2024).

SP is a member of the tachykinin family comprised of 11 amino acids, which is widely distributed in CNS and PNS (Assefa, 2023). SP mainly binds to the neurokinin 1 receptor (NK1R) on non-neuronal cells, including MSCs, osteoblasts and osteoclasts (Wang et al., 2009). Systemic injection of SP promotes the migration of CD29^+^ MSCs to the wound region, facilitating bone remodeling through the activation of Erk1/2 signaling pathway (Hong et al., 2009; Zhang et al., 2014). Previous research has indicated that local administration of SP in a rat mandibular DO model can accelerate bone remodeling and enhance MSC migration during osteogenesis (Zhang et al., 2014). Another research team revealed that gelatin microspheres containing CGRP or SP effectively accelerate bone formation, yet its therapeutic effects on human alveolar ridge defects still need further investigation (Chen et al., 2017).

Sema 3A is a neuroinformatic protein molecule widely expressed in various tissues and organs, exerting its influence via binding to two co-transmembrane receptors, neuropilin-1 (Nrp-1) and PlexinA family, which has drawn attention to its protective function in bone homeostasis (Wu et al., 2023a). It has been discovered that Sema 3A and Nrp1 binding inhibited osteoclast differentiation via the inhibition of the ITAM and RhoA signaling pathways and stimulated osteogenic differentiation through Wnt/β-catenin signaling pathway (Hayashi et al., 2012). It was revealed that Sema 3A is a fundamental positive regulator in human alveolar BMMSCs (hABMMSCs) osteogenic differentiation (Liu et al., 2018a). Studies from several independent groups revealed that Sema 3A may contribute to bone remodeling during orthodontic tooth movement (OTM) (Şen et al., 2021; Kamei et al., 2022; Mei et al., 2024). Şen et al. (2021) showed that human osteoblasts from alveolar bone responded to Sema 3A stimulation with a Rac1 and β-catenin-dependent induction of differentiation markers. Kamei et al. (2022) found that osteoblast-derived Sema 3A regulates alveolar bone remodeling during OTM by accelerating osteoblast calcification on the tension side and inhibiting osteoclastogenesis on the compression side. Besides, Mei et al. (2024) emphasized the important role of neurons-derived Sema 3A in loads-mediated bone formation during OTM. Exogenous Sema3A accelerated alveolar bone formation and the osteogenic differentiation of human periodontal ligament cells (hPDLCs) induced by mechanical overload (Mei et al., 2024).

Except for the nerve-related molecules mentioned above, FGF also plays a role in sensory nerves’ regulation of MSCs in jaws or, to be more specific, in incisor mesenchyme tissue homeostasis, and it serves as a bridge connecting sensory nerves to Gli1 + MSCs. It was revealed that sensory nerves activate Gli1 + MSCs via secreting Shh protein, which contribute to all mesenchymal derivatives in a mouse incisor model (Zhao et al., 2014). Recently, a study highlighted that FGF signaling is essential for the interaction between sensory nerves and the proximal mesenchyme of the incisor (Pei et al., 2023). Secreted from sensory neurons, FGF1 directly acts on Gli1+MSCs via binding with its receptor FGFR1 to sustain the mesenchyme tissue homeostasis, which influences MSC maintenance, odontoblast differentiation and migration (Pei et al., 2023). In terms of the mechanism, it was demonstrated that activated FGF/p-JNK signaling regulates mTOR-dependent autophagy activation in Gli1 + MSCs (Pei et al., 2023). This research, together with the study reported in 2024 (Pei et al., 2024), reveals FGF serves as a bridge connecting sensory nerves to Gli1 + MSCs and confirms the interaction between sensory nerves and Gli1 + MSCs existing in the alveolar bone area.

Sensory nerves are not the only type of nerves present in the craniofacial region. Sympathetic nerves are also involved in craniofacial bone homeostasis and repair. It has been reported in these years that sympathetic signaling regulates bone remodeling in a negative fashion (Elefteriou et al., 2014; Corr et al., 2017; Elefteriou, 2018). Chen et al. (2019) discovered that sensory nerve activation suppresses sympathetic tone and facilitates osteoblast proliferation and differentiation. Norepinephrine (NE), as the main neurotransmitter of the sympathetic nervous system, is synthesized from the amino acid tyrosine by the action of tyrosine hydroxylase (TH) (Elefteriou, 2018). Sympathetic nerves release NE to modulate the metabolism and function of osteoblasts and osteoclasts via α-adrenergic and β-adrenergic receptors (ARs), and NE participates in the regulation mainly through β2AR (Xiao et al., 2023). A study showed that propranolol, a β-AR blocker, effectively enhances implant osseointegration *in vivo*, facilitates proliferation of osteoblasts and promotes osteogenic differentiation of osteoblasts and MSCs (Wu et al., 2021). It was indicated that sympathetic denervation-induced MSC mobilization in rat mandibular DO is correlated with inhibition of MSC migration and osteogenic differentiation via NE/adrb3 signaling pathway (Du et al., 2014). Furthermore, Wu et al. (2016a) discovered that in the rat mandibular DO model, NE may upregulate the SDF-1 secretion by vascular endothelial cells through NE/adrb3/JNK pathway, thus inhibiting the MSC chemotaxis migration towards bone trabecular frontlines during bone regeneration.

#### 3.2.2 Neural regulation in suture/calvaria

The suture or calvaria is another position where nerves and MSCs are tightly connected and draws attention of researchers focusing on craniofacial bone homeostasis and repair. Nerve growth factor (NGF), vasoactive intestinal peptide (VIP) and molecules mentioned in the previous section (CGRP, SP and Sema 3A) have been discovered to be involved in the neural regulation of MSCs in suture/calvaria homeostasis and repair. NGF was previously considered as an indispensable neurotrophic factor related to sensory and sympathetic nerve growth (Hempstead et al., 1991). NGF binds to its high-affinity receptor, TrkA, which can be located on the surface of sensory nerves, and NGF is then transmitted by endosomes and experiences long-distance retrograde transport from the distal axon to the cell body of dorsal root ganglia (Skaper, 2017). A previous study demonstrated that the overexpression of TrkA, receptor of NGF, can promote Schwann-like cell differentiation of bone marrow stromal stem cells (Zheng et al., 2016). TrkA improves the survival and regenerative capacity of bone marrow stromal stem cells via the upregulation of the Erk/Bcl-2 pathway (Zheng et al., 2019). Tomlinson et al. (2016) showed that interruption of perichondrium-derived NGF expression or sensory nerve-associated TrkA activity prejudices long bone ossification. They then demonstrated an acute upregulation of NGF expression following long bone fracture in mice (Li et al., 2019). Research above is limited in long bone ossification and repair. This team then studied sensory nerves and NGF-TrkA signaling in mouse cranial bone healing. Abundant NGF is marked in mesenchymal cells of all patent cranial sutures, whereas NGF is scarce in fused posterofrontal suture (Meyers et al., 2020). Consistent with long bone (Li et al., 2019), NGF is sharply increased after calvarial bone injury. At early time points post-injury, NGF-expressing cell during this time were mainly PDGFRα + mesenchymal cells and F4/80 + macrophages, and bone-lining osteocalcin-positive osteoblasts were the major source of NGF during the later stages (Meyers et al., 2020). In addition, like the role of TrkA in long bone fracture, TrkA signaling also proved necessary for calvarial regeneration, and disruption of TrkA activity hindered re-innervation and delayed cranial bone defect healing (Meyers et al., 2020). Except for NGF-TrkA signaling pathway, this team also studied another NGF-related pathway. NGF-p75 signaling within mesenchymal lineage cells is crucial for cell migration and osteogenic differentiation during bone repair (Xu et al., 2022a). Additionally, Zhang et al. (2023b) proposed a neuromodulation approach to orchestrate critical-sized defects (CSDs) healing via the construction of engineered sensory nerves, and NGF released from sensory nerve-inductive scaffolds effectively induces sensory neuron differentiation and promotes CGRP secretion, leading to osteogenic differentiation of BMMSCs. The engineered sensory nerves accelerate osteogenesis via NGF-TrkA signaling and thus facilitate CSDs healing, providing an alternative solution for calvaria repair (Zhang et al., 2023b).

VIP is a neuropeptide which belongs to a glucagon/secretin superfamily, playing crucial roles in a broad spectrum of biological functions (Jiang et al., 2016). VIP binding with its receptor VPAC1 enhances BMMSCs osteogenic differentiation through Wnt/β-catenin signaling pathway *in vitro*, and VIP-conjugated functionalized hydrogel facilitates calvaria defect repair with increased osteogenesis and angiogenesis (Shi et al., 2020). In addition, CGRP, SP and Sema 3A also participate in calvarial repair. With recombinant lentiviral vector overexpressing CGRP, CGRP gene-modified BMMSCs were designed and found to be effective seed cells in tissue engineering to repair skull defects (Yu et al., 2019). Zhang et al. (2018) highlighted that intravenous SP attenuates inflammation both systemically and locally in calvarial injured mice. Intravenous SP can effectively facilitate the osteogenesis of MSCs and contribute to the calvarial CSD repair (Zhang et al., 2018). Besides, it was discovered that co-overexpression of Sema 3A and HIF1α promoted induced pluripotent stem cell-derived mesenchymal stem cells (iPSC-MSCs) osteogenesis and angiogenesis and bone regeneration (Li et al., 2020). They then applied Sema 3A-HIF1α co-overexpressed iPSC-MSCs seeded on HA scaffold to calvarial defect mouse model, and it turned out to be effective in bone repair (Li et al., 2020). Liu et al. (2016) showed that Sema 3A in BMMSCs is crucial for its potent osteogenic capacity, and transferring Sema 3A into adipose-derived stem cells (ASCs) partially reprograms the cells towards BMMSCs, thereby enhancing the osteogenic capacity. They further demonstrated that transplantation of Sema 3A infected ASCs significantly facilitate the bone regenerative capacities in the calvarial CSD model (Liu et al., 2016).

Similarly, except for sensory nerves and the nerve-related molecules, sympathetic nerves play a part in the regulation of MSCs in suture/calvaria homeostasis and repair. Li et al. (2022b) studied whether inhibition of sympathetic signaling assists in repair of CSDs. They designed a 3D printed hydrogel scaffold to imitate the microenvironment of bone regeneration, which is loaded with the calcium channel blocker nifedipine, thus decreasing the concentration of catecholamine and suppressing sympathetic activity in the bone defect region (Li et al., 2022b). They confirmed that this scaffold inhibited osteoclastogenesis, facilitated the migration and osteogenic differentiation of MSCs, and succeeded in rat cranial bone regeneration, implying the potential of sympathetic signaling inhibition for bone repair (Li et al., 2022b).

The main studies on neural regulation of MSCs in craniofacial bone homeostasis and repair are listed in Table 1. In brief, sensory nerves and sympathetic nerves with the nerve-related molecules collaborate in the regulation of MSCs in craniofacial bone homeostasis and repair. Sensory nerves are more inclined to promote osteogenesis while sympathetic nerves tend to regulate bone remodeling in a negative way.

**TABLE 1:** Studies concerning neural regulation of MSCs in craniofacial bone homeostasis and repair.

Location	Experimental model	Mediator (nerve/molecule/signaling pathway)	Effect	References
Jaws	IAN-denervated rat	IAN	Bone loss	Wu et al. (2016b)
	IAN-denervated rat		Reduction in incisor eruption and enamel defects	Hayano et al. (2018)
	IAN-denervated mouse/human; mouse/human MSC		Impaired osteogenesis	Tevlin et al. (2023)
	IAN-denervated mouse	IAN; PDGF-AA; OSM; PTH	Partial rescue of compromised bone repair	Jones et al. (2019)
	Mouse tooth extraction model	Sensory nerve; CGRP; SHH signaling	Stimulation of MSCs’ osteogenic differentiation	Pang et al. (2015)
	IAN-denervated rat	CGRP; Wnt signaling	Promoting osteogenesis	Liu et al. (2018b)
	*Ramp1* ^−/−^ mouse	CGRP-positive nerve	Induction of MPDL22 cells into hard tissue-forming cells	Miki et al. (2024)
	Rat mandible vertical osteotomy model	SP	Acceleration of bone remodeling and promoted MSC migration	Zhang et al. (2014)
	hABMMSC	Sema 3A	Promoting osteogenesis	Liu et al. (2018a)
	hPDLC; human osteoblast			Şen et al. (2021)
	Mouse OTM model			Kamei et al. (2022)
	Mouse OTM model; hPDLC			Mei et al. (2024)
	*Fgfr1* ^*fl/fl*^ *; Fgfr2* ^*fl/fl*^ mouse	IAN; FGF/p-JNK signaling	mTOR-dependent autophagy in MSCs	Pei et al. (2023)
	Rat mandibular DO model	Sympathetic nerve; NE/adrb3 signaling	Inhibition of MSC migration and bone regeneration	Du et al. (2014), Wu et al. (2016a)
Suture/Calvaria	Mouse calvarial defect model	Sensory nerve; NGF-TrkA signaling	Promoting bone defect healing	Meyers et al. (2020)
	Rat calvarial defect model			Zhang et al. (2023b)
	Mouse calvarial defect model	Sensory nerve; NGF-p75 signaling	Promoting cell migration and osteogenic differentiation	Xu et al. (2022a)
	Rat calvarial defect model; Rat BMMSC	VIP; Wnt/β-catenin signaling	Stimulation of BMMSCs osteogenic differentiation and bone repair	Shi et al. (2020)
	Rat calvarial defect model; Rat BMMSC	CGRP	Promoting bone repair	Yu et al. (2019)
	Mouse calvarial defect model	SP	Facilitating osteogenesis and bone repair	Zhang et al. (2018)
	Mouse calvarial defect model; iPSC-MSCs	Sema 3A	Promoting bone repair	Li et al. (2020)
	ASC; Rat calvarial defect model	Sema 3A	Reprogramming ASCs towards BMMSCs and enhancing osteogenesis	Liu et al. (2016)
	BMMSC; Rat calvarial defect model	Sympathetic nerve	Inhibition for bone repair	Li et al. (2022b)

Abbreviations: adrb3, adrenoceptor beta 3; ASC, adipose-derived stem cell; BMMSC, bone marrow mesenchymal stem cell; CGRP, calcitonin gene-related peptide; DO, distraction osteogenesis; FGF, fibroblast growth factor; hABMMSC, human alveolar bone marrow mesenchymal stem cell; hPDLC, human periodontal ligament cell; IAN, inferior alveolar nerve; iPSC-MSC, induced pluripotent stem cell-derived mesenchymal stem cell; MPDL22, mouse periodontal ligament clones; MSC, mesenchymal stem cell; mTOR, mammalian target of rapamycin; NE, norepinephrine; NGF, nerve growth factor; OSM, oncostatin M; OTM, orthodontic tooth movement; PDGF-AA, platelet-derived growth factor-AA; p-JNK, phosphorylated c-Jun N-terminal kinase; PTH, parathyroid hormone; Ramp1, receptor activity-modifying protein 1; Sema 3A, semaphorin 3A; SHH, sonic hedgehog; SP, substance P; TrkA, tropomyosin receptor kinase A; VIP, vasoactive intestinal peptide.

## 4 Conclusion and perspective

In this review article, we summarized several MSCs located in craniofacial bones, including Gli1 + cells, Axin2 + cells, Ctsk + cells, Prx1 + cells, LepR + cells, etc. Then we discussed studies on the role of nervous system in craniofacial bones via the regulation of varied MSCs, primarily focused on bone development, homeostasis and repair. The regulation mainly depends on nerves with a series of molecules, such as NGF, CGRP, FGF, and NE, and several signaling pathways. As we can see, neural regulation of MSCs in the skeletal tissue is a hot topic of research currently. Reports in the last decade on nerve innervation and its participation in bone development and metabolism in craniofacial bones have been mentioned in the section “Neural regulation in craniofacial bone.” Nevertheless, several aspects in this field remain to be completed.

First, studies concerning neural regulation of MSCs with specific markers are limited. The recent advance in craniofacial bone MSCs is reported in the second part “MSCs in craniofacial bone,” and varied markers labeling MSC in jaws, TMJ and sutures have been identified. The third part “Neural regulation of MSCs in craniofacial bone” is based on the second part, introducing recent studies on the neural regulation of MSCs. However, the connection between nerves and MSCs with specific markers mentioned in the second part has not been verified completely. We highlighted Gli1 + cells which are widely studied in craniofacial biology in the third part of this review article, and emphasized that Gli1 + cells in mouse incisor and molar mesenchyme were demonstrated to be regulated by sensory nerves, in which FGF serves as the bridge (Pei et al., 2023; Pei et al., 2024). Besides, Yu et al. (2021) verified that suture Gli1 + MSCs with resorbable biomaterials support regeneration of cranial sutures in craniosynostosis mouse model, and dura mater cells contribute to Gli1 + cells in suture regeneration. Although no direct evidence of neural relevance in this process was found, sensory or sympathetic nerves may intervene in the regulation of Gli1 + MSCs, considering the dura mater is richly innervated by afferent nerve fibers from the trigeminal ganglion and by sympathetic fibers (Lv et al., 2014). In addition to Gli1 + MSCs, neural regulation of other MSC populations is worth further exploration. Second, most of the current reports did not further elucidate the exact mechanisms mediating neural regulation of MSCs in craniofacial bones. The majority of research explored the mechanisms merely on the level of specific nerves or molecules, while few studies figured out the signaling pathways underlying neural regulation of MSCs in craniofacial bone. Third, since the role of nerve fibers in the regulation of MSC in craniofacial bones has been gradually explored, research on the inverse regulation from stem cells on nerves may be valuable, which is relatively inadequate. Outside the area of craniofacial bones, nerve-stem cell crosstalk in skin regeneration and diseases has been studied. Signals from skin stem cell lineages specify a niche during development, inducing peripheral nerve fibers to innervate varied stem cell populations, and nerve fibers coordinate with skin stem cells to support tissue homeostasis and regeneration in adults (Peng et al., 2022). Fourth, previous research on the clinical application of craniofacial bone homeostasis and repair mainly focused on MSCs alone. Further exploration of neural regulation of MSCs may assist in modulation and treatment in the craniofacial bone diseases or injuries, such as the healing of craniosynostosis and the resolution of calvarial CSD repair.
